# Harnessing nanotechnology: Transforming ocular drug delivery with advanced hydrogel systems

**DOI:** 10.1002/btm2.70099

**Published:** 2026-06-04

**Authors:** Huidi Cui, Hao Wu, Bizhu Zhao, Yameng Zhao, Chengzhi Zhang, Mingxuan Wang, Jiehao Zhang, Kexin Wang, Yiwen Wang, Zongming Song, Ye Tao

**Affiliations:** ^1^ Department of Ophthalmology Henan Eye Hospital, Henan Provincial People's Hospital, People's Hospital of Zhengzhou University Zhengzhou China; ^2^ Zhengzhou University Henan Medical College Zhengzhou University School of Medicine Zhengzhou China

**Keywords:** eye disease, nanofiber hydrogels, ocular drug delivery, ophthalmic applications

## Abstract

Ocular drug delivery faces tremendous challenges in clinical practice. The eyeball possesses sophisticated anatomical and physiological characteristics that uniquely influence the pharmacokinetics of delivered drugs. This complexity necessitates innovative solutions to ensure effective drug delivery. Conventional ocular drug administration routes (topical, intravitreal, and systemic) each pose unique limitations. With the advancements of biomaterials science and medical engineering technology, nanofiber hydrogels have garnered significant attention, primarily represented by self‐assembled peptide‐based hydrogels and cellulose nanofiber‐based hydrogels. They can be tailored to have precise physical and chemical properties, enabling controlled release of drugs and enhanced biocompatibility. Furthermore, their nanofibrous structure mimics the extracellular matrix, promoting cell adhesion and tissue regeneration. This review introduces advanced manufacturing techniques which are capable of precisely modifying the properties of nanofiber hydrogels to meet specific therapeutic needs. The distinctive advantages of nanofiber hydrogels are elaborated in detail, including their ability to enhance drug penetration, provide sustained release, and reduce systemic toxicity. We also delve into the therapeutic applications, potential limitations, and developmental perspectives of nanofiber hydrogels. Preclinical studies have validated their efficacy in treating ophthalmologic conditions such as age‐related macular degeneration, bacterial keratitis, ocular alkali burns, and non‐infectious uveitis. However, translating these laboratory findings into clinical applications remains limited, primarily due to significant challenges in human trials, including species‐specific responses, the complexity of human biology, and the safety of nanofiber hydrogels. Future refinements in fabrication techniques and rigorous safety assessments are necessary to revolutionize the clinical application of nanofiber hydrogels.

## Graphical abstract

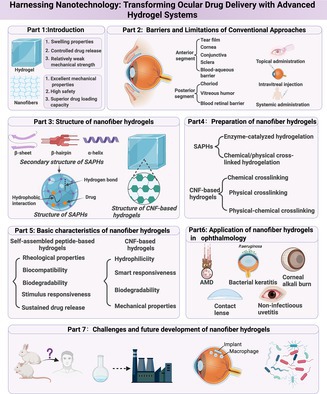

**Abbreviations**
EO: ethylene oxide
FDA: Food and Drug Administration
Fmoc‐FF: N‐fluorenylmethoxycarbonyl diphenylalanine
FTIR: Fourier Transform Infrared Spectroscopy
H&E: hematoxylin and eosin
IL‐1: interleukin‐1
IL‐6: interleukin‐6
IL‐1B: interleukin‐1 beta
NDA: new drug application
NF‐κB: nuclear factor‐kappa B
nVAMD: neovascular age‐related macular degeneration
PA: palmitic acid
SH: Sulfhydryl Group
TEMPO: (2,2,6,6‐Tetramethylpiperidin‐1‐yl)oxyl
Th1: T helper 1 cell
Th17: T helper 17 cell
TNF‐α: tumor necrosis factor‐alpha
XRD: X‐ray diffraction

**Translational Impact Statements**

Nanofiber hydrogel systems offer a paradigm shift in ocular drug delivery, enabling sustained therapeutic release that extends effective treatment time up to threefold while reducing injection frequency. This technology significantly lowers risks like endophthalmitis and retinal toxicity associated with frequent intravitreal injections. With demonstrated preclinical success in age‐related macular degeneration, keratitis, and uveitis, and potential for smart contact lenses, these biocompatible hydrogels promise to enhance patient compliance, minimize systemic side effects, and revolutionize the management of vision‐threatening diseases. Clinical translation hinges on scalable manufacturing and rigorous long‐term safety validation.

## 1 INTRODUCTION

Ocular diseases pose a tremendous threat to the quality of patients' lives and potentially contribute to vision impairment or even blindness. Thus far, more than 250 million individuals are affected by visual impairment around the world.^1^ It is predicted that approximately 115 million people may go blind by 2050 without improved therapies. In clinical practice, ocular drug delivery is confronted with formidable challenges due to various anatomical and physiological barriers. Topical ocular drug delivery methods for treating anterior segment diseases have a relatively low bioavailability due to a high clearance rate. Posterior segment diseases are commonly treated with intravitreal administration of therapeutic agents, which may lead to retinal toxicity, increased intraocular pressure, and increased risk of endophthalmitis.^2^ In this context, there is an urgent need to develop novel therapeutic approaches for ocular diseases. To address this critical challenge, researchers are working on innovating drug delivery systems that can enhance the bioavailability and safety of ophthalmic treatment. In the past decades, various strategies to improve the bioavailability of drugs have been extensively explored. Among these, drug delivery systems such as nanomaterials, liposomes, and hydrogels have gained attention for their abilities to enhance the penetration and retention of drugs. Particularly, hydrogels stand out among these novel drug delivery systems, possessing high biocompatibility, the ability to accommodate and release drugs, and stimulant responsiveness. Hydrogels are composed of crosslinked polymers, which endow them with swelling properties when immersed in an aqueous environment. Compared with traditional drug delivery systems, hydrogels afford the controlled release of drugs with better biocompatibility for biological tissue.^3^ Meanwhile, the integration of advanced materials like hydrogels with traditional medicine presents a unique opportunity to bridge ancient wisdom with modern therapy, further expanding their therapeutic potential.^4^ However, the mechanical strength of hydrogels made only by molecular‐level crosslinking is relatively weak. To overcome this limitation and further enhance the performance of ocular drug delivery systems, scientists have turned to combining hydrogels with other advanced biomaterials. For instance, nanoparticle‐hydrogel composites formed by combining nanoparticle systems with cationic peptide hydrogels can be utilized for efficient ocular drug delivery.^5^ Furthermore, by incorporating dexamethasone (Dex) and the reactive oxygen species (ROS) scavenger cerium‐based metal organic framework into thermosensitive hydrogels, anti‐inflammatory and antioxidant effects are achieved for the treatment of endotoxin‐induced uveitis.^6^ Beyond drug delivery, innovative hydrogel designs are also tackling the challenge of rapid ocular clearance. Recently, researchers successfully achieved the remineralization of early enamel caries and the inhibition of bacterial biofilms by constructing carboxymethyl chitosan‐lysozyme nanogels loaded with antibacterial drugs (tea polyphenols, silver nitrate, and chlorhexidine) and amorphous calcium phosphate, providing a reference for the development of bifunctional nanomaterials with both antibacterial and tissue repair functions.^7^ Similarly, an injectable lubricative copolymer demonstrated long‐lasting relief in a dry eye model by adhering to the ocular surface, showcasing the potential of advanced material design to overcome bioavailability limitations.^8^ Encouragingly, nanofibers have emerged as promising nanomaterials due to their excellent mechanical properties and high safety. Nanofibers possess a large surface area and superior drug‐loading capacity, thus, minimizing the need for frequent administration. It is increasingly recognized that combining nanofibers and hydrogels can synergistically improve their functions while attenuating their potential drawbacks. Currently, the synthesis of nanofiber hydrogels by mixing nanofibers into hydrogels represents an innovative research realm. Its logical approach is to incorporate nanofiber as a filling material into a larger hydrogel network. From this, the nanofiber hydrogels have suitable mechanical properties, adhesion, ductility, and the ability to mimic the microstructure of the extracellular matrix (ECM) and the microenvironment of the cell. Advantageously, these characteristics make nanofiber hydrogels highly versatile and widely applicable in multiple tissues.

Thus far, the nanofiber hydrogels have been used in different aspects of clinical practice, including soft tissue reconstruction, accelerating diabetic wound healing, bio‐microactuator application and wound dressing.^9^, ^10^, ^11^, ^12^ Previous studies have demonstrated that combining electrospun poly(allyl‐caprolactone) fibers with a hyaluronic acid (HA) hydrogel network can generate a composite material that mimics the microstructure and mechanical properties of the ECM in soft tissue. This composite material has the properties of high porosity and promotes the infiltration of host cells. Experimental studies indicate that this injectable nanofiber composite hydrogel can promote the tissue regeneration reaction and ultimately achieve durable soft tissue repair outcomes.^12^ In addition, a nanofiber hydrogel core‐shell scaffold with a three‐dimensional multilayer patterned structure has been developed to promote diabetic wound healing and accelerate angiogenesis. This scaffold features a remarkably porous architecture that facilitates cell adhesion and infiltration, thereby accelerating tissue regeneration.^11^ Owing to its open pore structure and high specific surface area, it can load and release drugs by adjusting external environmental conditions, which makes it a promising candidate for drug delivery. *However, the current research landscape remains fragmented*. While numerous studies exist on hydrogels or nanofibers individually, a systematic comparison and comprehensive review focusing specifically on nanofiber hydrogels for ocular drug delivery are notably lacking. For example, existing literature often fails to directly contrast the design principles, material properties, and therapeutic advantages of self‐assembled peptide‐based hydrogels (SAPHs) versus cellulose nanofiber (CNF)‐based hydrogels. In the meantime, there is a lack of critical discussion on the common and overall challenges that hinder the clinical transformation of these advanced materials, especially in terms of regulatory pathways, long‐term safety, and scalable manufacturing.

Therefore, to supplement the current research and enrich the content of this field, this review aims to provide a comprehensive and critical analysis of nanofiber hydrogels for ocular drug delivery. We first systematically elaborate on the chemical structures and fabrication methods of these advanced materials. Furthermore, their biological, physical, and physicochemical characteristics will be described in detail, and their prospects in ophthalmology will be thoroughly evaluated. The article will also delve into specific therapeutic applications of nanofiber hydrogels in treating various ophthalmologic conditions and outline the key challenges associated with their clinical translation. With continued advancements in nanofiber hydrogels research, ocular drug delivery systems are poised to be optimized to better meet the practical needs of patients.

## 2 PHYSIOLOGICAL BARRIERS AND LIMITATIONS OF CONVENTIONAL APPROACHES

**FIGURE 1:**
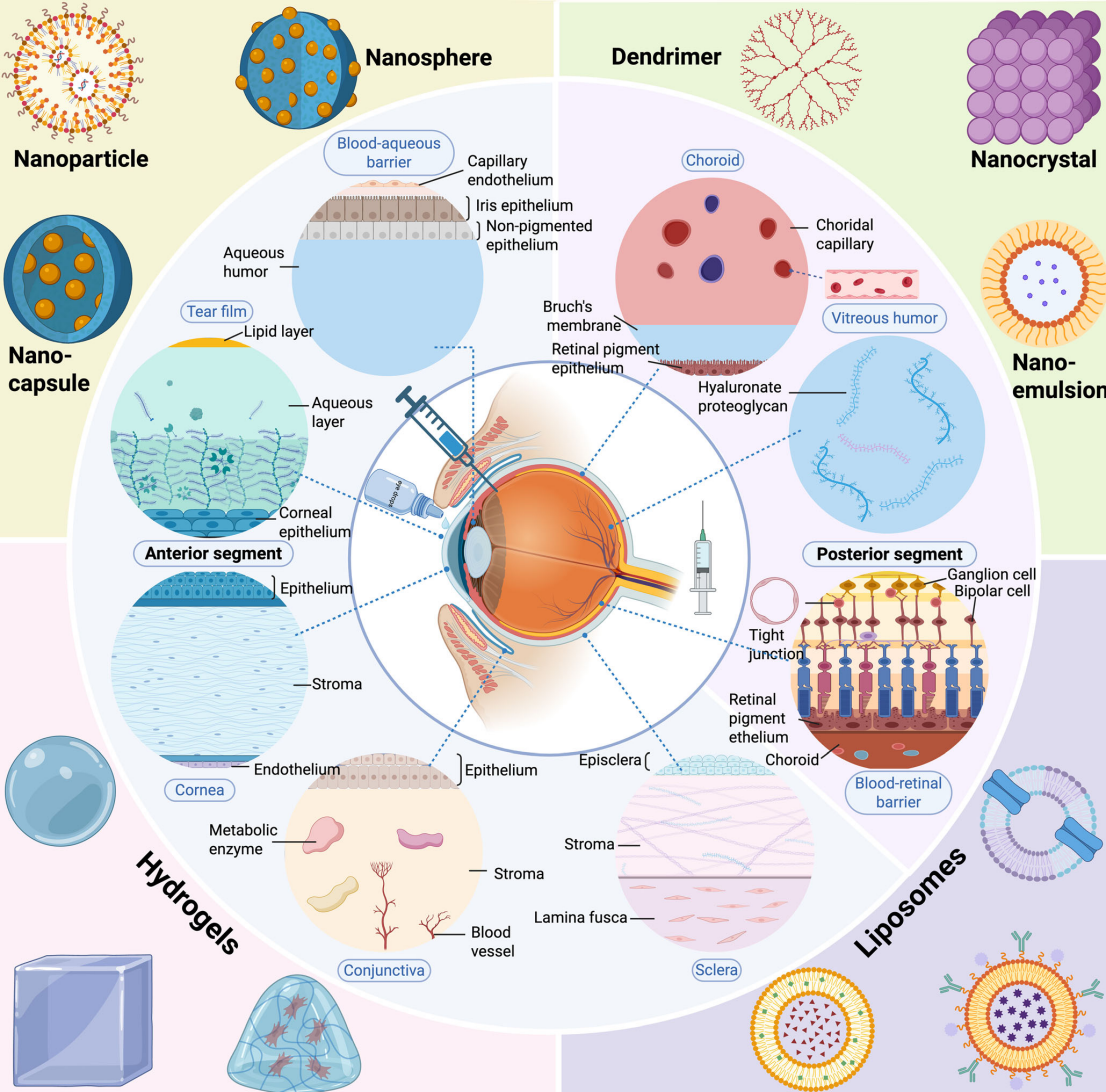
The key physiological barriers against ocular drug delivery, and various nanocarriers commonly used to overcome these barriers. The anterior segment barriers encompass the tear film, cornea, iris, sclera, and blood‐aqueous barrier. The posterior segment barriers include the choroid, vitreous humor, and blood‐retinal barrier.

As one of the most complicated sensory organs, the eye has unique physiological and anatomical structures, with which it can connect, regulate, and coordinate visual function.^1^ In general, the eyeball is segmented into the anterior and posterior segments. The anterior chamber comprises the cornea, sclera, anterior chamber, aqueous humor, and lens, while the posterior chamber starts behind the lens and ends at the optic nerve, which includes the choroid, retina, vitreous humor, and optic nerve. In the anterior segment, there are the tear film, cornea, conjunctiva, sclera, and blood‐aqueous humor barrier (BAB) serving as critical barriers. As a thin and transparent liquid layer, the tear film is composed of three layers: the lipid layer on the surface, the water layer in the middle, and the mucus layer in the inner. The first two layers act as a barrier to hydrophilic and hydrophobic drugs, while the mucus layer attracts or repels drugs through electrostatic interactions, protecting the eye from external stimuli and pathogens. The cornea serves as the primary barrier preventing external substances from entering the anterior chamber of the eyeball. Its structure, composed of the epithelium, stroma, and endothelium from the outermost to the innermost layer, collectively functions as a biological barrier. These layers jointly limit drug penetration into the anterior segment of the eye, thereby maintaining ocular integrity and functions. Located on the back surface of the eyelid and outside the cornea, the conjunctiva is a mucous membrane composed of the vascularized epithelium and the inner stromal layer, which is involved in the formation and maintenance of the tear film and protects the ocular surface from environmental pathogens. As an opaque and hard sheath, the sclera wraps around the surface of the eyeball. It consists of the episcleral layer, the stroma layer, and the lamina fusca. Its thickness is a key factor affecting the passage of drugs through the sclera. The BAB, composed of tight junctions in the non‐pigmented ciliary epithelium and the endothelium of iris blood vessels, prevents solutes from entering the intraocular environment.^13^ For the posterior segment of the eye, the vitreous barrier and blood‐retinal barrier (BRB) act as the primary barriers against exogenous substances. The main solute of vitreous fluid is hyaluronic proteoglycan, which impacts the rate of drug movement to retinal tissue.^14^ As the most principal barrier in the posterior segment of the eye, the BRB is formed by internal and external components. The inner BRB is composed of tight connections between endothelial cells in retinal capillaries, while the outer BRB comprises tight connections between retinal pigment epithelial cells.^15^ The BRB regulates the penetration efficiency of elements from systemic circulation to the retina.^16^ In addition to these two main barriers, the choroid also blocks light from penetrating the sclera into the eye to ensure clear imaging. Consequently, drug transport to target ocular tissues is highly restricted (Figure 1).

These barriers fundamentally constrain the efficacy of traditional delivery methods. Topical administration, such as eye drops, while non‐invasive and patient‐preferred, suffers from extremely low bioavailability for both anterior and posterior segment diseases due to the rapid nasolacrimal drainage, aqueous fluid secretion, decreased corneal epithelial permeability, systemic absorption, and BAB.^17^ Intravitreal injection bypasses some obstacles and directly delivers the drug to the vitreous body, but the vitreoretinal barrier can prevent large molecules from entering the retinal pigment epithelium (RPE), making it difficult to reach an effective concentration. At the same time, this is an invasive surgery, which carries the risks of endophthalmitis, retinal detachment, and elevated intraocular pressure.^18^ The need for frequent injections (e.g., monthly for anti‐vascular endothelial growth factor [VEGF] therapy) also leads to poor patient compliance and a high economic burden. Moreover, systemic administration is severely limited by the BRB and drug‐binding proteins in plasma, often requiring high doses that result in systemic toxicity without achieving therapeutic intraocular concentrations.^19^, ^20^

To address these challenges, advanced drug delivery platforms such as nanofiber hydrogels have gained prominence for ocular applications, such as contact lenses, patches, and local delivery systems.^21^, ^22^ Among these systems, SAPHs and CNF‐based hydrogels have attracted extensive research interest in ocular drug delivery systems due to their exceptional biocompatibility, tunable physicochemical properties, and biomimetic structural advantages, which demonstrate satisfactory biocompatibility and enable long‐term controlled drug release.^10^, ^23^ Other traditional systems, such as those based on synthetic polymers, may face limitations in biocompatibility, potential toxicity of degradation products, or lack of biomimetic properties. Thus, SAPHs and CNF‐based hydrogels demonstrate superior potential for both research and practical applications in ocular drug delivery. Herein, we intend to introduce the two categories of nanofiber hydrogels, including their structures, preparation methods, characteristics, and applications in ophthalmology.

## 3 STRUCTURE OF NANOFIBER HYDROGELS

### 3.1 Structure of SAPHs

The formation of SAPHs begins with the initial self‐assembly of peptide molecules into specific secondary structures. These secondary structures then further aggregate to form nanofibers, which serve as the fundamental building blocks of the hydrogel network.^24^ Due to the differences in conformations, the secondary structure of polypeptides can be divided into β‐sheet structure, β‐hairpin, and α‐helix structure. The type of secondary structure of the polypeptide is determined by the amino acid sequence and ultimately has a pivotal influence on the properties of the hydrogel. Therefore, in order to make the final hydrogel have excellent properties, researchers should pay special attention to the design of the secondary structure of the polypeptide (Table 1). Herein, we mainly focus on the two categories of secondary structures, β‐sheet and α‐helix.

**TABLE 1:** Comparative analysis of self‐assembled peptide‐based hydrogels (SAPHs) and cellulose nanofiber (CNF)‐based hydrogels for ocular drug delivery.

Aspect	SAPHs	CNF‐based hydrogels
Fabrication ease	More difficult	Easier
Mechanical strength	Softer and more tunable; highly dependent on the peptide sequence and assembly structure	Superior mechanical strength and toughness, exhibiting high toughness, compressive strength and elasticity
Drug release profile	Highly tunable and responsive, allowing for precise control over sustained‐release kinetics in response to specific biological stimuli	Primarily diffusion‐controlled; can be engineered for stimuli‐responsive release
Biocompatibility	Excellent	Remarkable
Biodegradability	Degraded by protease	Degraded by cellulase

If a self‐assembling polypeptide is to be designed to be amphiphilic, the polar and non‐polar residues in the polypeptide should be placed alternately. This is exactly what happens when a self‐assembled peptide forms a β‐sheet structure. The sequence of the peptide chain that can self‐assemble to form a β‐sheet structure is generally the (XZZX)n sequence, in which X represents a non‐polar amino acid and Z represents a polar amino acid. Hydrophilic and hydrophobic groups are alternately arranged and distributed on both sides of the sheet, in which the hydrophobic residues are buried in the inner side and the hydrophilic residues are on the outside. As for the other major secondary structure, the α‐helix structure, the most common peptide sequence is made up of repeated characteristic heptamer (a b c d e f g)_n_.^25^ In general, the positions of a and d are typically occupied by two amino acids with a high helix tendency, such as alanine and leucine. At the same time, the positions of e and g are usually charged amino acid residues, while the positions of b, c, and f are often occupied by polar amino residues.

### 3.2 Structure of CNF‐based hydrogels

Cellulose, the most abundant natural polymer on earth, serves as the fundamental building block of CNF‐based hydrogels. It is a linear polysaccharide composed of repeating β‐D‐glucose units, characterized by its biodegradability, renewability, and biocompatibility, making it widely applicable in the fabrication of hydrogels and aerogels.^26^ Unlike conventional polymer‐based hydrogels, CNF‐based hydrogels are structured as three‐dimensional nanofiber networks formed through extensive hydrogen bonding between CNFs—each with a nanoscale diameter and micrometer‐scale length.^27^, ^28^ This nanofibrous architecture is highly porous and continuous, providing ample space for water storage and thus conferring exceptional water absorption and retention capabilities. Moreover, the intrinsic mechanical properties of CNFs—such as high strength and stiffness (with a Young's modulus up to 138 GPa, comparable to steel and Kevlar)—are effectively transferred to the macroscopic hydrogel (Table 1). As a result, CNF‐based hydrogels combine high‐water content with remarkable mechanical strength and toughness, overcoming the typical mechanical limitations of traditional hydrogels and positioning them as promising materials for applications in bone repair, wound healing, and ocular drug delivery.^9^, ^27^, ^29^ This highly biomimetic nanofiber structure also enables it to simulate the microscopic environment of the ECM very well, further enhancing their potential in a range of biomedical fields.

## 4 PREPARATION OF NANOFIBER HYDROGELS

### 4.1 Preparation of SAPHs

SAPHs can be prepared via different methods. In this section, we outline two methods for preparing SAPHs: (i) enzyme‐catalyzed gelation and (ii) gelation induced by chemical or physical crosslinking.

#### 4.1.1 Enzyme‐catalyzed hydrogelation

The preparation of small molecular enzymatic hydrogels consists of three steps: (1) the precursor is converted into small molecular hydrogels through the enzyme bond breaking; (2) the derived small molecular hydrogels are self‐assembled into nanofibers; (3) the nanofibers are wound to form a hydrogel matrix.^24^ Notably, the SAPHs formed by self‐assembly of small molecules show an orderly arrangement within randomly winding nanofibers, and the molecular order of nanofibers is adjustable through simple structural modifications. In addition, spatiotemporal hydrogel assembly is achievable in specific cellular environments through the selective exposure of enzyme cleavage sites in lysosomes, using acid phosphatase as a guiding enzyme (Table 1). For instance, researchers have discovered that tyrosinase can trigger the gel–sol transformation of small molecule hydrogels.^30^ The dephosphorylation of Ac‐YYYpY‐OMe (Ac stands for carboxyl group, Y for tyrosine, p for phosphate group, and OMe for methoxy group) generates Ac‐YYYY OMe, which can self‐assemble into a hydrogel. This hydrogel is responsive to tyrosinase and can be degraded under the catalysis of tyrosinase. Due to the overexpression of tyrosinase in malignant melanoma, this enzyme‐responsive hydrogel has been reported to be able to encapsulate anti‐cancer drugs for the treatment of malignant melanoma.

From a translational perspective, the scalability of enzyme‐catalyzed hydrogelation for Good Manufacturing Practice (GMP) presents a nuanced picture. Its strengths lie in high specificity and mild reaction conditions, favoring product purity and consistency. However, scale‐up is hampered by three key challenges: the high cost of pharmaceutical‐grade enzymes, the need for precise control over gelation kinetics, and the thermolabile nature of enzymes, which necessitates complex and costly aseptic manufacturing. These are ultimately solvable engineering hurdles that ongoing development is expected to overcome.

#### 4.1.2 Chemical/physical crosslinked hydrogelation

Chemical crosslinking and physical crosslinking are two important methods for preparing SAPHs. As for the former, the mechanical strength of SAPHs is enhanced by covalent chemical crosslinking. Side chains of amino acids, such as thiol and catechol groups, undergo covalent crosslinking by oxidation.^31^ Thiol groups allow reversible crosslinking through disulfide bonds and are always used for controlled drug release. Catechol is partially crosslinked by oxidation‐induced stabilization of peptide fibers.^32^

For example, Seow et al.^33^ used cysteine‐containing short peptides as assembly units and took advantage of the property that cysteine can form disulfide bonds to prepare disulfide bond crosslinked transparent hydrogels. This hydrogel has been reported to have high hardness and shape fidelity, which is conducive to transplantation operations with tweezers during surgery. On the other hand, the physical crosslinking method provides versatility in designing peptide fiber networks via reversible supramolecular binding. Electrostatic interactions, metal coordination bonds between metal ions, amino acid side chains, hydrophobic interactions and molecular recognition in β‐sheet fibers collectively contribute to physical crosslinking, allowing the formation of stable and structured hydrogels.^34^ Researchers utilized the electrostatic interaction between the negatively charged dipeptide Fmoc‐FF and the positively charged PLL‐SH (PLL stands for polylysine) to enhance the entanglement between assembled peptide nanofibers, thereby obtaining injectable hydrogels.^35^ Therefore, injectable hydrogels based on the integration of self‐assembling small peptides and biological macromolecules possess immense potential for transformative biomedical and translational applications.

### 4.2 Preparation of CNF‐based hydrogels

In general, the preparation methods of CNF‐based hydrogels mainly include chemical, physical, and physical–chemical crosslinking methods. Properties of CNF hydrogels vary dramatically with different crosslinking methods. Herein, we summarize these three preparation methods.

#### 4.2.1 Chemical crosslinking

Chemical crosslinking refers to the method of forming a hydrogel network through covalent crosslinking interactions, which endows hydrogels with excellent mechanical properties and structural stability. Free radical polymerization, Schiff base reaction, and Diels‐Alder (DA) reaction are three important chemical reactions involved in the preparation of CNF‐based hydrogels through the chemical crosslinking method. For instance, the researchers involved the synthesis of a thermally reversible hydrogel by using the DA “click” reaction in an aqueous medium. This thermally reversible nanocellulose hydrogel was prepared by first modifying TEMPO‐oxidized CNFs with furfurylamine to introduce furan groups, then crosslinking them with a water‐soluble bismaleimide via the DA reaction at 60°C in water. The resulting hydrogel could be decrosslinked through retro‐DA at 95°C and reformed upon cooling, demonstrating full thermal reversibility.^36^

Free radical polymerization is a polymer reaction triggered by an initiator under certain conditions, and then the polymer reacts with a good deal of monomers to form an extended polymer chain structure.^37^ Free radical polymerization can regulate the properties of hydrogels by changing the monomers and initiators, such as high elasticity, self‐healing, and intelligent response.^38^ Additionally, this method facilitates rapid fabrication of hydrogels with well‐defined network structures. For instance, scientists prepared CNF hydrogels grafted with poly(acrylic acid–co‐acrylamide) through free radical polymerization. After adding 10% CNF, the mechanical strength of the hydrogel increased by 13 times and the maximum deformation increased by two times.^39^ Nevertheless, free radical polymerization is difficult to control, and it is easy to form an uneven network structure. Conversely, the radiation crosslinking not only has a rapid crosslinking speed but also has the capacity to form a homogeneous crosslinking network structure. For example, a nanocellulose‐based hydrogel was synthesized through the γ radiation crosslinking method, using polyacrylic acid solution and graphene oxide as raw material to mix with CNF.^40^ This hydrogel demonstrates potent water absorption capacity without toxicity to human skin.

Schiff base reaction refers to the condensation reaction of aldehydes or ketones with compounds (such as amines) containing ‐NH_2_ groups to produce imine or methylamine organic compounds. Especially, in the absence of external stimulation, the Schiff base reaction can give CNF‐based hydrogels perfect self‐healing properties. Due to the presence of abundant hydroxyl groups in CNF, it can be chemically modified to participate in the Schiff base reaction. For example, after being oxidized by sodium periodate, nanocellulose can be transformed into dialdehyde nanocellulose, which then reacts with amino compounds to form nanocellulose hydrogel with remarkable self‐healing performance. In addition to endowing CNF‐based hydrogels with self‐healing properties, the Schiff base reaction also improves the mechanical properties of nanocellulose hydrogels. For instance, researchers have found that the stress of gelatin hydrogel after adding dialdehyde functionalized CNF was 3.398 MPa, while that of gelatin hydrogel after adding CNF was 0.192 MPa, and that of pure gelatin hydrogel was 0.058 MPa.^41^ Accordingly, the Schiff base reaction improves efficiently the mechanical properties of hydrogel. What is more, the Schiff base reaction can alter the structure of the hydrogel network, thereby affecting the porosity and swelling properties of hydrogel.^42^ In general, the self‐healing properties, mechanical properties, and swelling properties of nanocellulose hydrogels can be adjusted by the Schiff base reaction.

DA reaction is a reversible covalent crosslinking reaction between conjugated dienes and dienophiles.^43^ It takes place under mild conditions, requires no catalyst or initiator, and produces harmless byproducts. DA reaction is often used to prepare in situ materials, notably hydrogels for drug delivery and tissue engineering.^36^ For instance, researchers synthesized biocompatible thermally reversible hydrogels by the DA reaction of water‐based nanocellulose fibers containing furan with water‐soluble bismaleimide in aqueous medium.^44^ Additionally, Heidarian et al.^45^ prepared a double crosslinked network by combining DA reactions and hydrazone bonds. The results showed that DA reactions could improve the structural integrity and mechanical strength of hydrogels, while hydrazone bonds endowed hydrogels with self‐healing properties. The produced hydrogels hold promise as a candidate for cartilage tissue engineering.

#### 4.2.2 Physical crosslinking

Physical crosslinking refers to the method of forming hydrogels through non‐covalent interactions, but the mechanical properties of hydrogels prepared by this crosslinking method are weaker than those of the chemical crosslinking method. Due to the large number of hydroxyl groups in the molecular structure of cellulose, a network structure can be formed through hydrogen bonding, and then cellulose‐based hydrogels can be formed by physical crosslinking. Hydrogen bonds impart hydrogels with outstanding biocompatibility and certain self‐healing properties.^46^ In addition to hydrogen bonding, ionic crosslinking endows hydrogels with special responsiveness, while host–guest interactions give hydrogels excellent mechanical properties. In the subsequent sections, the application of these three interactions in the preparation of CNF‐based hydrogels will be introduced in detail.

Hydrogen bonds are very common in biological systems and are indispensable in the activities of biological tissue. Their reversibility also endows hydrogels with self‐healing properties. However, the weak interaction properties of hydrogen bond crosslinking result in unstable hydrogel structures. Whereas cellulose contains rich hydroxyl groups, facilitating the formation of hydrogels with strong hydrogen bond networks.^47^ CNFs have been used to form strong networks to prepare CNF‐based hydrogels with excellent mechanical properties, and the CNF‐based hydrogels are seen as viable wound dressing.^48^ Additionally, investigators have established a novel stretchable, electrically conductive, and self‐healing hydrogel‐based strain sensor using the poly(vinyl alcohol) (PVA), graphene (GN), borax crosslinking agent, and CNF. In this sensor, the hydrogen bonds formed between GN–CNF, PVA, and borax are extremely crucial for its self‐healing properties.^49^

As a common method for preparing hydrogels, ionic crosslinking refers to the process of linking polymer chains by reacting ionic groups on the polymer with ions of opposite charge, thereby fabricating adjustable hydrogels. The hydrogels prepared by ion crosslinking can transform according to environmental factors such as pH or ionic strength. This responsiveness can be modulated by changing the type and concentration of ions in the crosslinking process.^50^, ^51^ Nevertheless, other ions in the reaction system may interfere with ion crosslinking, thus affecting the properties of the prepared hydrogels. To enable ionic crosslinking, we can introduce charged functional groups such as sulfonic acid or carboxyl groups to nanocellulose. Subsequently, the nanocellulose is obtained by sulfuric acid hydrolysis and the TEMPO oxidation method. For instance, researchers prepared self‐supporting CNF hydrogels by ionic crosslinking Zn^2+^ with TEMPO‐oxidized bagasse CNF, whose structure and compressive strength were regulated by ionic bond strength.^52^

Host–guest interaction refers to the process of the combination of large ring subject and small ring guest through non‐covalent interaction to form supramolecules with specific functions.^53^ Typically, nanocellulose itself cannot participate in the host–guest interaction. Hence, it is necessary to attach the host or guest molecules involved in the reaction to nanocellulose. Cyclodextrin (CD), as an amphiphilic polymer, can form inclusion compounds through the host–guest interaction, which is often used in responsive drug delivery and biomedical engineering. Owing to the reversible nature of supramolecular bonding, hydrogels formed through host–guest interaction between CD and guest molecules exhibit excellent mechanical properties and self‐healing abilities.^54^ For example, in previous studies, researchers modified cellulose nanocrystals with adamantane (Ad) as the guest molecule and introduced the main molecule β‐CD, which formed the host–guest interaction, and finally designed a supramolecular composite hydrogel.^55^ The experimental results show that the supramolecular composite hydrogel exhibits enhanced mechanical properties due to the host–guest interaction between Ad and β‐CD. It is worth noting that the highly specific nature of host–guest interaction allows us to tailor the structure of the hydrogels network by adjusting the host or guest molecules.

#### 4.2.3 Physical–chemical crosslinking

Physical–chemical crosslinking combines the advantages of physical crosslinking and chemical crosslinking, thus giving nanocellulose hydrogels excellent mechanical properties, self‐healing ability, and swelling function. For instance, researchers have prepared the double‐crosslinked nanocellulose hydrogels by the physical–chemical crosslinking method, which showed superior mechanical properties compared with single‐crosslinked nanocellulose hydrogels. These double‐crosslinked nanocellulose hydrogels could withstand a compressive strength of more than 450 kPa and 90% compressive strain without cracking. In addition, these nanocellulose hydrogels have perfect optical transparency and excellent swelling properties, which make them promising candidates in the realm of ophthalmic biomaterials. Researchers have also produced a double crosslinked nanocellulose‐borax‐PVA hydrogel via a physical–chemical crosslinking method. The hydrogel exhibits high flexibility and shape controllability due to the penetration of the flexible PVA network through the rigid nanocellulose network. Moreover, the borax crosslinker endows the hydrogel with remarkable self‐healing ability and high ionic conductivity.^56^ Moreover, this hydrogel can serve as a green platform to integrate with antibiotics to create non‐toxic antibacterial materials, which may offer new possibilities for ophthalmic biomaterials.

Beyond the preparation methods mentioned above, advanced processing techniques such as electrospinning and 3D bioprinting have been widely utilized to fabricate nanofiber hydrogel scaffolds with precise microstructures. Take electrospinning technology as an example; it has been widely employed to fabricate micro/nanofibers from various polymers—including hydrogel‐forming materials—for a broad range of biomedical applications.^57^ In a typical electrospinning setup, a high‐voltage power supply is connected to a metal needle, while a collector is grounded. A syringe pump feeds polymer precursor solution through the needle at a controlled rate. Under applied voltage, a droplet forms at the needle tip and is stretched by electrostatic repulsion. Once this repulsion exceeds the droplet's surface tension, micro/nanofibers are ejected toward the grounded collector.^58^ During the flight of the liquid jet, the solvent evaporates, leaving behind solid fibers that accumulate on the collector.

## 5 BASIC CHARACTERISTICS OF NANOFIBER HYDROGELS

### 5.1 Characteristics of SAPHs

The distinctive preparation methods and network structure of SAPHs endow them with exceptional rheological characteristics. As a result, they exhibit excellent injectable properties, making them highly promising candidates for drug delivery applications.^59^ Moreover, the peptide composition in the SAPHs imparts them with outstanding biocompatibility and biodegradability. Due to these advantages, they have been extensively studied and applied in various biomedical fields. Notably, SAPHs also demonstrate stimuli‐responsive behavior and controlled drug release capabilities, further expanding their potential in targeted therapeutic strategies.

**FIGURE 2:**
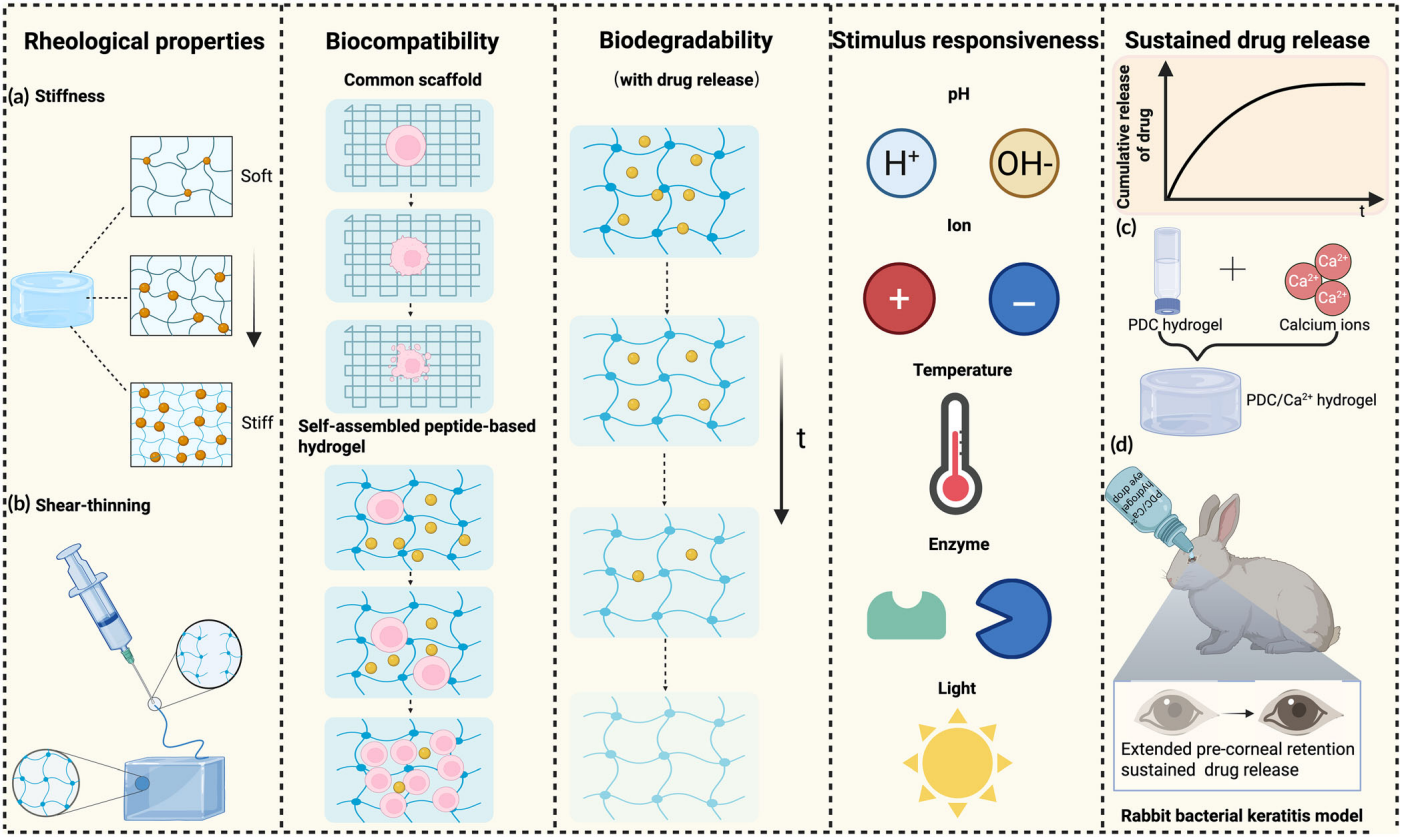
Characteristics of self‐assembled peptide‐based hydrogel. The figure is segmented into five primary characteristics, each vital to the functionality and compatibility of nanofiber hydrogels, including rheological properties, biocompatibility, biodegradability, stimulus responsiveness, sustained drug release. PDC, peptide–drug conjugate.

Rheological properties are the primary factors affecting biological function and medical application. The characteristic parameters of rheological properties generally include storage modulus (*G*′), loss modulus (*G*″) and loss factor (tan *δ*).^60^ The storage modulus, reflecting gel stiffness, critically impacts biomedical performance (Figure 2a). For instance, hydrogels with low stiffness, exhibiting a *G*′ value on the order of 60–100 Pa, can promote drug diffusion and dynamic cell adhesion, making them efficient for drug delivery.^61^ While the FEFEFKFK peptide‐based SAPH exhibits a high *G*′ value of 0.8–20 kPa, making it ideal for application as a tissue scaffold.^62^ Additionally, shear‐thinning is another important rheological characteristic of SAPHs (Figure 2b). It defines the phenomenon that viscoelastic materials such as hydrogels change from a highly viscous solid state to a low viscosity liquid state at high shear rates.^63^ When such hydrogels are subjected to shear force, the *G*′ value decreases greatly, and when the shear force disappears, the *G*′ value recovers again. Hence, the shear‐thinning property endows hydrogels with the ability to repair themselves. However, systematic reports on the swelling ratios of various SAPHs are scarce, necessitating future work on their quantitative characterization to advance clinical translation. The shear‐thinning and self‐healing properties of SAPHs provide potential applications as injectable drug and cell delivery vehicles. For instance, using self‐assembly dynamics and shear‐thinning repair dynamics, researchers designed uniformly encapsulated C3H0t1/2 stem cells within MAX‐8 hydrogels. When subjected to a 1000% strain, these hydrogels transition into a liquid state and immediately revert to their original form when the strain is removed. The unique properties allow them to be injected using syringes and precisely delivered to target biological sites for tissue repair, which may have profound implications for eye drug delivery.^64^ This is especially advantageous for posterior segment diseases, where precise and sustained drug delivery is critical.

Moreover, the peptide composition in SAPHs gives them excellent biocompatibility and biodegradability, offering a higher possibility for their wide application in various fields of biomedicine. The cells were encapsulated within (PA)‐attached NAPVSIPQKKK (PA‐NK) hydrogels via the self‐assembly of peptides (PA‐NK) in the presence of the cells through a peptide solution. The viability of the encapsulated cells remained close to 100% within 48 h, and the cells retained their normal morphology. Furthermore, researchers inoculated cells on the surface of a PA‐NK hydrogel to further assess the hydrogel's cytotoxicity. These cells exhibited robust adhesion and growth within 24 h.^65^ Additionally, SAPHs can be responsive to different stimuli, such as pH, ions, temperature, and enzyme, further expanding their potential applications^66^ (Table 1). For instance, pH triggers gelation as a result of protonation or deprotonation of amines or carboxyl groups in the peptide chain, resulting in a transition between the hydrogel and solution states. Furthermore, by incorporating the thermosensitive polymer poly(*N*‐isopropylacrylamide) (PNIPAM) into the self‐assembled three‐dimensional network of peptide nanofibrils, this system exhibits a sol state below 33°C and a gel state conversely. This thermosensitive hydrogel provides a novel biomaterial for intelligent drug delivery and tissue engineering applications.^67^ More importantly, in terms of drug delivery, SAPHs can achieve sustained drug release. Researchers chose to pair a small self‐assembled peptide Gly‐Phy‐Phy‐Tyr‐ASP (GFFYD) with chloramphenicol to form a peptide–drug conjugate (PDC), which could then assemble itself to form a hydrogel.^68^ It is found that this PDC hydrogel realize sustained drug release under the mediation of Ca^2+^ (Figure 2c). Meanwhile, to evaluate in vivo therapeutic efficacy, a rabbit bacterial keratitis model was employed. PDC and PDC/Ca^2+^ hydrogels demonstrated minimal inflammatory responses compared to PBS and commercial eye drops, suggesting their potential as promising ocular drug delivery systems (Figure 2d). The ability to sustain drug release over extended periods is particularly beneficial for chronic ocular conditions, reducing the need for frequent administrations and improving patient adherence.

### 5.2 Characteristics of CNF‐based hydrogels

**FIGURE 3:**
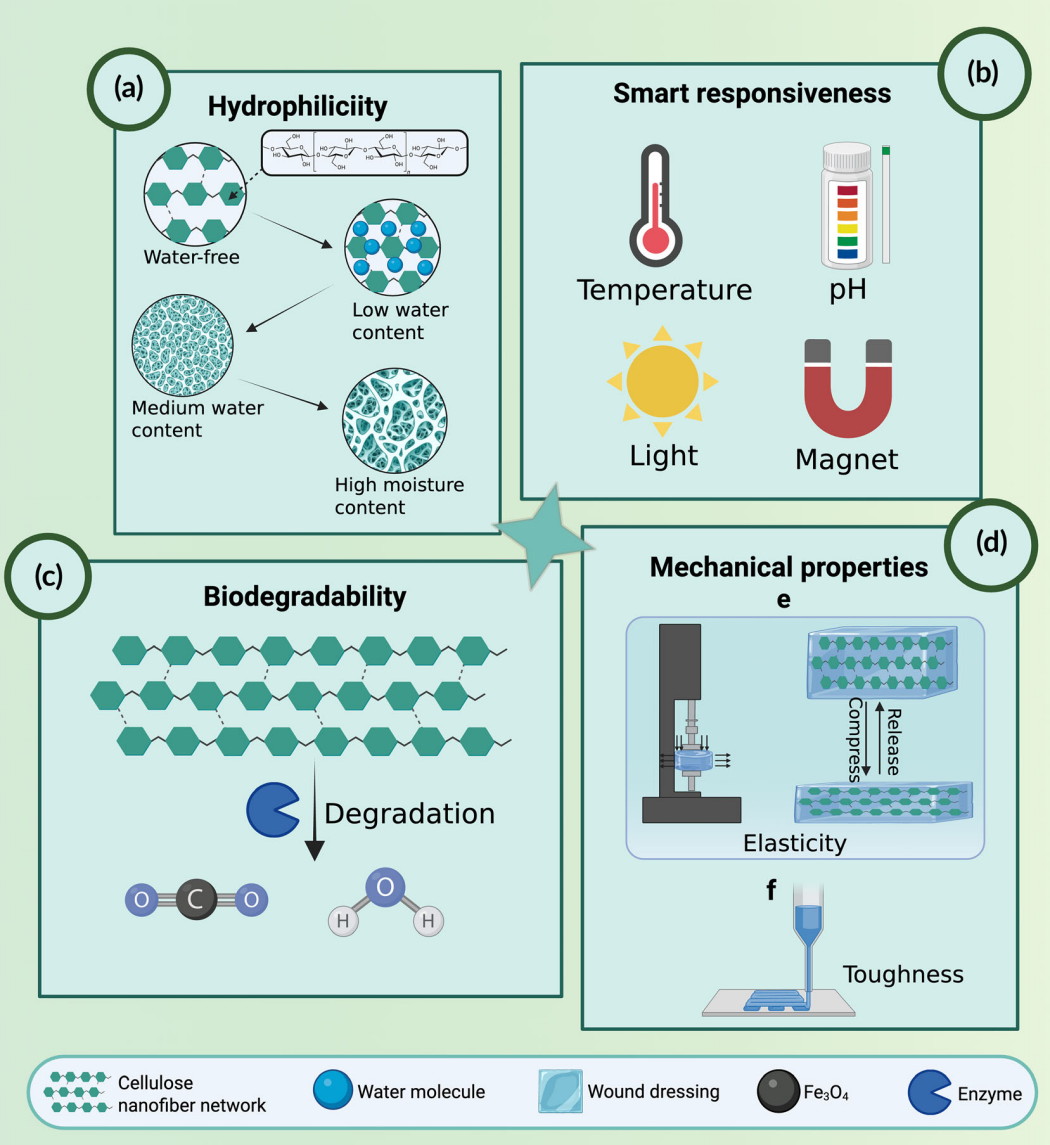
Characteristics of cellulose nanofiber‐based hydrogels. (a) hydrophilicity, (b) smart responsiveness, (c) biodegradability, and (d) mechanical properties. The segment of smart responsiveness encompasses its practical applications, while the mechanical properties section includes elasticity (e) and toughness (f).

CNF‐based hydrogels have unique three‐dimensional nanofiber networks and excellent physical and chemical properties. This makes them ideal materials for ophthalmic applications, including drug delivery, artificial corneas, and contact lenses.^69^, ^70^ Their high‐water content and soft mechanical properties closely mimic the native ocular environment, enhancing comfort and compatibility. Primarily, CNF‐based hydrogels exhibit perfect hydrophilicity (Figure 3a). Cellulose is a natural polysaccharide with a large number of hydroxyl (‐OH) groups in its molecular chain. These hydroxyl groups exhibit strong polarity and form hydrogen bonds with water molecules, imparting potent hydrophilicity to cellulose and its derivatives. In CNF‐based hydrogels, CNFs are connected to each other by hydrogen bonds to form a three‐dimensional network structure, which further enhances their capacity to absorb water molecules. This high hydrophilicity is advantageous for maintaining corneal hydration and promoting tear film stability, which is essential for dry eye management and ocular surface health. Moreover, CNF‐based hydrogels respond intelligently to temperature, pH, light, and reduction, demonstrating significant promise for smart drug delivery, wound dressings, and water purification^71^, ^72^ (Figure 3b). For instance, by incorporating CNF into a *N*‐isopropylacrylamide (NIPAM) hydrogel and simultaneously introducing disulfide bonds, a CNF/NIPAM hydrogel that exhibits dual redox and temperature responsiveness has been established. The novel hydrogel with intelligent response properties enables controlled release of drugs under specific conditions, while showing no obvious cytotoxicity.^72^ Such responsiveness can be harnessed for triggered drug release in response to ocular inflammation or infection, improving therapeutic outcomes. Cellulose, a macromolecular polysaccharide composed of glucose, is the most abundant natural polymer on Earth. Owing to its inherent degradability, CNF‐based hydrogels have garnered significant research attention. When enzymatically degraded, cellulose breaks down into smaller, benign molecules such as carbon dioxide, water, and inorganic compounds, which can be readily metabolized or excreted by the body (Figure 3c). While in vitro enzymatic hydrolysis in buffers is slow, the in vivo environment—rich in enzymes like lysozyme and ROS—likely accelerates degradation by attacking the amorphous regions of cellulose (Table 1). This kinetic difference must be considered to tailor the material's residence time for drug delivery or tissue engineering. This combination of natural origin, predictable clearance, and reduced risk of long‐term accumulation underscores the high biocompatibility of CNF‐based hydrogels for intraocular applications. Apart from the aforementioned characteristics, adding CNFs to hydrogel systems is a feasible way to optimize their mechanical properties (Figure 3d). As excellent nanoscale building blocks, CNFs exhibit high strength and stiffness,^73^ enabling the design of mechanically robust hydrogels with tailored toughness and fracture resistance for demanding applications like tissue scaffolds and flexible electronics.^74^, ^75^ Furthermore, the inherent nanofiber network and hydrogen bonding between CNFs and water molecules impart remarkable elasticity to CNF‐based hydrogels, allowing them to compress under stress and fully recover their original shape upon release. Recently, a highly elastic hydrated CNF‐based hydrogel derived from natural wood demonstrated exceptional resilience, recovering its original shape without structural damage after 10,000 compression cycles. This was achieved through a process involving NaOH/NaSO_3_ treatment to partially dissolve lignin and hemicellulose, followed by freeze‐drying and rehydration.^76^ Critically, CNF‐based hydrogels offer superior toughness compared to traditional hydrogels. For instance, triple‐network CNF hydrogels exhibit exceptionally high toughness (15.12 ± 0.14 MJ/m^3^), enabling them to withstand large deformations without fracture.^77^ This combination of elasticity, toughness, and recovery makes them particularly suitable for applications requiring repeated deformation, such as wearable sensors for real‐time human motion monitoring and human–machine interaction devices. The mechanical robustness and elasticity of CNF hydrogels are advantageous for contact lenses and corneal implants, ensuring durability and comfort during prolonged wear.

Collectively, the superior hydrophilicity, intelligent responsiveness, inherent biodegradability, and excellent mechanical properties of CNF‐based hydrogels position them as highly promising candidates for a wide array of biomedical applications, particularly in ophthalmology, where sustained, comfortable, and responsive drug delivery is paramount.

## 6 APPLICATIONS OF NANOFIBER HYDROGELS IN OPHTHALMOLOGIC PRACTICE

The selected applications in this section exemplify three key advantages of nanofiber hydrogels in ophthalmology: (1) as long‐acting intravitreal depots for chronic posterior segment diseases (age‐related macular degeneration [AMD], uveitis), (2) as enhanced topical delivery systems for anterior segment diseases (keratitis, alkali burns, and self‐delivery), and (3) as integrated biocompatible devices (smart contact lenses) (Figure 5). This framework highlights the rationale behind the chosen examples.

### 6.1 Age‐related macular degeneration

**FIGURE 4:**
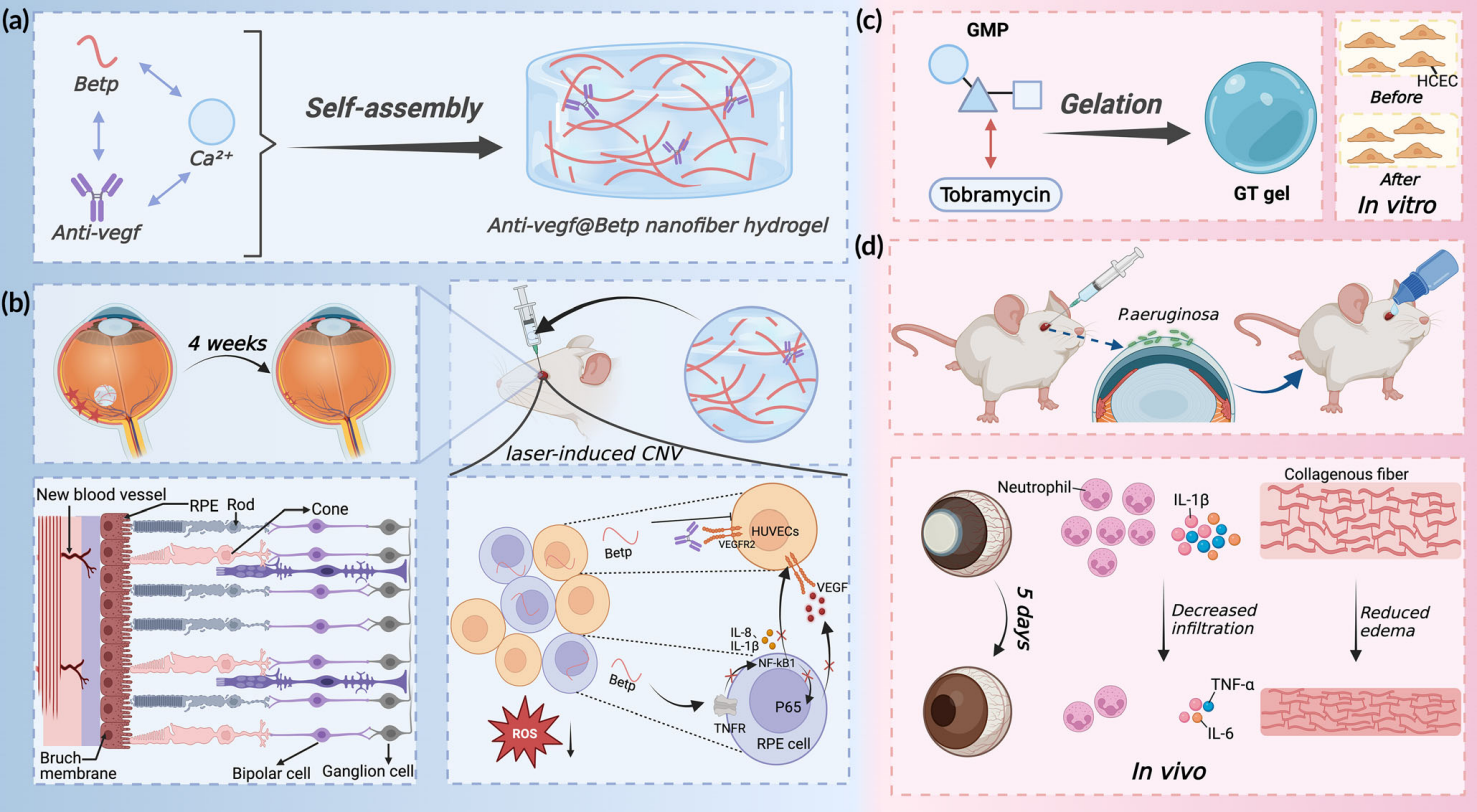
Applications of nanofiber hydrogels in ophthalmology. (a) Preparation of anti‐vegf@BetP nanofiber hydrogel; (b) The effects, method of administration and mechanism of anti‐vegf@BetP nanofiber hydrogel; (c) Preparation of guanosine‐5′‐monophosphate disodium salt/tobramycin (GT) gel and its effect of in vitro tests; (d) Establishment of mouse models of bacterial keratitis, GT gel's administration route and the efficacy of GT gel in vivo tests. BetP, betamethasone phosphate; CNV, choroidal neovascularization; GMP, guanosine‐5′‐monophosphate disodium salt; HCEC, human corneal epithelial cells; ROS, reactive oxygen species; RPE, retinal pigment epithelium.

AMD is a retinopathy mainly affecting the macular area, and acts as an important cause of visual impairment in the elderly population. Typically, AMD is classified as early stage and late stage. Early‐stage AMD is characterized by the emergence of glass membrane warts and RPE abnormalities. Late‐stage AMD primarily manifests in two forms: dry or atrophic, and wet or neovascular.^78^ Both types can lead to significant and irreversible loss of central vision. Wet AMD, also known as neovascular AMD, is characterized by the development of new abnormal blood vessels under the macula during choroidal neovascularization (CNV). These microangiopathies subsequently result in macular edema, intraocular hemorrhage retinal hypoxia, and eventually fibrosis scar.^79^ Several lines of evidence have shown that VEGF plays a crucial role in the development of CNV. Currently, the preferred treatment for CNV is the intravitreal injection of anti‐VEGF drugs like ranibizumab.^80^ However, frequent intravitreal injections may lead to adverse outcomes such as glaucoma, endophthalmitis, and retinal detachment.^81^ Moreover, frequent intravitreal injections can substantially reduce patient compliance and increase economic burden. Anti‐VEGF drugs have limited efficacy against fibrous scars and may even compromise the long‐term viability of the retinal neurons. Thus, there is an urgent need to develop new drug delivery systems for treating wet AMD. In search of effective therapies, an injectable supramolecular nanofiber hydrogel has been designed (Figure 4a,b). This hydrogel was synthesized by mixing disodium betamethasone phosphate (BetP), a steroid drug, with a calcium chloride solution in a 1:1 molar ratio.^82^ During the process of hydrogel formation, anti‐VEGF drugs (ranibizumab) were incorporated into the BetP solution. Subsequently, the researchers injected the nanofiber hydrogel into the vitreous of murine models with laser‐induced CNV. The hydrogel effectively inhibits the production of inflammatory factors such as TNF‐α and NF‐κB p65. Moreover, it significantly mitigates the intracellular ROS level, alleviating oxidative damage in RPE cells. In particular, it effectively suppresses VEGF expression, inhibits vascular leakage and reduces fibrous scar formation in AMD mouse models. Compared with traditional anti‐VEGF therapy, this nanofiber hydrogel can extend the effective treatment time by three times; this prolonged release profile substantially reduces the frequency of intravitreal administration and minimizes associated complications. It also reduces the occurrence of complications from frequent intraocular injections and minimizes long‐term anti‐VEGF drug effects on the retina and choroid (Table 2). Given its simple preparation process and the clinical approval of all productive reagents, this hydrogel may hold potential significance for clinical translation. Furthermore, studies have demonstrated that incorporating conductive polyaniline (PANi) into the nanofiber phase of scaffolds enables fabrication of conductive hydrogel/fiber composite scaffolds.^75^ These constructs closely recapitulate the inner and outer collagenous layers of the human retinal Bruch's membrane, offering the closest structural mimicry to the native tissue. This biomimetic platform provides an optimal system for assessing electrophysiological cues regulating polarized RPE behavior and elucidating the influence of specific signals on RPE cells. Electrochemical characterization revealed high electrical conductivity (1.84 ± 0.21 S/cm) and Young's modulus (2.66 ± 0.33 MPa), confirming superior mechanical properties. Notably, human RPE (hRPE) cells exhibited >70% viability after 3 and 7 days of culture, validating excellent biocompatibility of the hydrogel/fiber scaffolds. In a nutshell, the therapeutic efficacy of this injectable supramolecular nanofiber hydrogel mainly stems from its shear‐thinning and self‐healing properties, which permit gentle intravitreal injection, support fast structural recovery, and ultimately ensure sustained drug release at the target site. The hybrid hydrogel/fiber composite demonstrates translational potential across multiple domains such as retinal tissue engineering, regenerative medicine, in vitro modeling of retinal pathologies, and drug discovery for ocular diseases.

**TABLE 2:** Summary of selected preclinical and clinical studies on hydrogel‐based systems for ocular therapy.

Disease	Hydrogel system	Study type	Model/stage	Outcomes	Trial ID/references
Age‐related macular degeneration (AMD)	NT‐503 implant	Clinical	nVAMD patients (terminated at Phase II)	Well‐tolerated but did not meet primary efficacy endpoint	NCT03144999
	Anti‐vegf@BetP nanofiber hydrogel	Preclinical	Laser‐induced CNV model in mice	Prolonged effective treatment time (three‐fold vs. traditional anti‐VEGF), suppressed inflammation/neovascularization, reduced side effects, inhibited fibrous scar formation	82
Bacterial keratitis	GT gel	Preclinical	*Pseudomonas aeruginosa*‐induced bacterial keratitis in mice	Achieved the restoration of transparency faster, decreased corneal inflammatory infiltration, and collagen edema.	83
	Peptide–drug supramolecular hydrogel	Preclinical	*Escherichia coli*‐induced bacterial keratitis in rabbits	Excellent biocompatibility, controllable drug release, and powerful antibacterial ability significantly prolong the retention time of drugs on the ocular surface	68
Neurotrophic keratitis	Noveome's ST‐266	Clinical	Phase II trial	Investigating safety and efficacy for treating neurotrophic keratitis	NCT04553432
Ocular wound healing	PUTK/RH patch	Preclinical	Alkali burn‐induced model in rats	Good transparency, mechanical properties, and antioxidant capacity and potential for HAM alternative	84
Non‐infectious uveitis (NIU)	Dexamethasone‐loaded Supramolecular Hydrogel	Preclinical	EAU model established in rats	Reduced adverse reactions, relieved inflammation, excellent biocompatibility, and controlled drug release	85
Eye inflammation and pain after ophthalmic surgery	Dextenza®	Clinical	FDA approved	A single insertion provides 30 days of corticosteroid therapy for ocular inflammation and pain; demonstrates the clinical viability of sustained‐release hydrogel platforms	NDA 208742
Self‐delivery system for ophthalmic drugs	Dex‐SA supramolecular hydrogel	Preclinical	Rabbit models	Prolonged precorneal retention time and higher bioavailability	86

Abbreviations: CNV, choroidal neovascularization; Dex‐SA, succinated dexamethasone; EAU, experimental autoimmune uveitis; GT, guanosine‐5′‐monophosphate disodium salt/tobramycin; HAM, human amnion membranes; PUTK, polyurethane; RH, reactive oxygen species‐clearing hydrogel; VEGF, vascular endothelial growth factor.

Admittedly, AMD is a refractory chronic condition posing significant therapeutic challenges. There is still a long way to exploit effective and safe nanofiber hydrogels that can be translated into clinical applications. More in‐depth mechanism studies are urgently needed to validate whether this delivery strategy can provide long‐term therapeutic effects in clinical practice.

### 6.2 Bacterial keratitis

Bacterial keratitis, a severe ocular infection that can cause serious visual impairment, is associated with etiological factors such as contact lens use, corneal trauma, and prior eye surgery.^87^ Topical administration of broad‐spectrum antibiotics is still the prime clinical option for treating bacterial keratitis. However, traditional ophthalmic drugs usually have poor bioavailability due to the unique anatomical and physiological barriers of the eye. Encouragingly, hydrogels have been utilized to deliver ophthalmologic drugs owing to their prolonged retention on the ocular surface, high moisturizing properties, and excellent biocompatibility. Traditional anti‐inflammatory eye gels primarily rely on sticky polymer substrates or thermosensitive polymers to encapsulate antibiotic drugs. These synthetic and non‐degradable polymers can irritate the eye tissues.^12^

On the other hand, supramolecular hydrogels composed of natural small molecules through non‐covalent interactions have demonstrated considerable advantages in treating bacterial keratitis. These hydrogels not only possess excellent rheological properties, but also ensure excellent biocompatibility and biodegradability, making them suitable for establishing injectable or spray formulations. It is well known that tobramycin (TOB), the first‐line antibacterial agent for bacterial keratitis, offers perfect biocompatibility, but it suffers from poor bioavailability as an eye drop. Therefore, a novel all‐small molecule supramolecular hydrogel composed of guanosine‐5′‐monophosphate disodium salt (GMP) and TOB has been exploited, in which GMP is assembled into guanosine quartet nanofibers by forming hydrogen bonds and π–π stacking; TOB crosslinks nanofibers with multiple phosphate groups to form gel networks through ionic interactions.^83^ Researchers conducted both in vitro experiments and animal studies to evaluate the therapeutic effects of the GMP/TOB hydrogel (GT gel). The GT gel was applied on the ocular surface of a *Pseudomonas aeruginosa*‐induced bacterial keratitis mice model (Figure 4c,d). Firstly, no significant toxic effect was observed either in human corneal epithelial cells (HCEC) cultures or in mouse cornea, verifying its high biocompatibility. Secondly, compared with the TOB eye drop treatment group, the mice in the GT gel treatment group achieved the restoration of transparency within a shorter period of time, and the mice treated with GT gel had fewer infiltrated neutrophils in the infected cornea, which should be attributed to GT gel's ability to consistently release TOB. Moreover, the expression of pro‐inflammatory cytokines such as IL‐1β and IL‐6 was significantly inhibited in the infected lesion after GT gel treatment, and the results of H&E staining also indicated that corneal inflammatory infiltration and corneal collagen fiber edema were significantly reduced (Table 2). The superior therapeutic efficacy of the GT gel over conventional eye drops is attributed to its ability to form a stable network on the corneal surface, enabling sustained TOB release. This resulted in a local bactericidal concentration maintained for over 25 h, overcoming the rapid precorneal clearance characteristic of eye drops. The sustained‐release profile enhanced ocular bioavailability, leading to higher bacterial clearance, which was corroborated by a one‐order‐of‐magnitude reduction compared to the free TOB eye drop group in bacterial load. In addition, the exceptional rheological properties of the GT gel, including shear‐thinning and thixotropy, enable its application as an injectable or sprayable formulation, adapting seamlessly to the ocular surface and providing prolonged drug retention. Another peptide‐based hydrogel has been developed for the treatment of bacterial keratitis.^68^ Covalent coupling of small peptides (GFFYD) with chloramphenicol gives PDCs the ability to self‐assemble, thereby establishing a peptide‐based hydrogel. Simultaneously, the elasticity of the hydrogel is regulated by adding calcium ions. This hydrogel exhibits potent antibacterial activity against both gram‐positive and gram‐negative bacteria in vitro, while remaining non‐toxic to corneal epithelial cells. To verify the hydrogel's therapeutic effect in vivo, experimenters established a rabbit bacterial keratitis model induced by *Escherichia coli*, and further tests demonstrate that the hydrogel achieves longer precorneal retention without ocular irritation and maintains sustained antibacterial activity (Table 2). Similarly, the peptide‐based hydrogel achieved longer precorneal retention and sustained antibacterial activity, owing to its stable nanofibrous structure that modulates drug release kinetics. This peptide‐based hydrogel possesses good biocompatibility and outstanding rheological properties, which give it significant advantages in treating bacterial keratitis.

In summary, nanofiber hydrogels may represent an innovative biomaterial that may prospectively replace existing drug delivery systems for treating bacterial keratitis in the future.

### 6.3 Ocular wound healing

Ocular chemical injuries are common clinical ophthalmic emergencies, among which alkali burn is the most serious one. Alkali burn occurs when alkaline chemicals come into contact with the eyelids, conjunctiva surface, and cornea, and is characterized by a persistent inflammatory response. The pathologic process of ocular alkali burn can be divided into the acute stage, the concomitant stage, and the repair stage. The longer the alkali stays in the eye, the more serious the damage it causes. Thus, timely intervention in the acute phase is crucial. During the acute phase, oxidative stress emerges as a key contributor to pathogenesis. Inflammation promotes ROS expression in the ocular surface, leading to epithelial cell injury and even apoptosis. The excessive ROS further amplify the pro‐inflammatory signal, including IL‐1, IL‐6, and TNF‐α, thus, forming a vicious cycle. Therefore, the elimination of overexpressed ROS may act as an effective therapeutic strategy for ocular chemical injuries. It is acknowledged that 8oxo2′‐deoxyguanosine (8‐oxo‐dG) eye drops are a classic therapeutic drug against inflammation. However, its short ocular surface retention, inefficient delivery, and frequent dosing requirements compromise patient compliance and increase the risk of systemic toxicity. Polyurethanes (PUs) are easily processed into various forms, such as fibers and hydrogels, demonstrating excellent biocompatibility and mechanical properties.^88^ PU/silk hybrid electrospun nanofiber scaffolds exhibit optimized physical and mechanical properties and are conducive to corneal epithelial differentiation.^89^

In a recent study, an in situ‐formed HA hydrogel, crosslinked by blue light‐induced thiol‐ene reaction, exhibited significant re‐epithelialization promotion and anti‐inflammatory effects on a rat corneal alkali burn model.^90^ However, HA alone cannot promote wound healing in severe chemical injuries. To address this limitation, a recently developed patch combining ROS‐clearing hydrogel (RH) with ROS‐clearing polyurethane (PUTK) containing copper sulfonate bonds has been used to treat corneal alkali burns in a rat model.^84^ In vitro experiments showed that the PUTK/RH patch exhibited higher transparency compared with human amnion membranes (HAM) group and PUTK film. Although the researchers did not report the value of light transmittance, they attributed the improvement in the transparency of the hydrogel sheet to its high‐water content (>90%) and refractive index similar to water (~1.33), thereby minimizing light scattering to the greatest extent. Moreover, the PUTK/RH patch demonstrated remarkable mechanical robustness, antioxidative capacity, and biological stability a in HCECs culture. In a rat model induced by alkali burn, the PUTK/RH patch significantly inhibited inflammation and enhanced the regeneration of corneal epithelium, representing a promising alternative to human amniotic membranes (Table 2). However, further research is essential to evaluate its long‐term scar‐inhibition capability and capacity to reconstruct the ocular surface.

### 6.4 Electronic smart contact lenses

Electronics on contact lenses have garnered significant research attention for their potential applications in ocular diagnosis and augmented reality. However, most of the existing contact lens devices are made of gas impermeable substrates, electronic components, and interfacial adhesive layers. These gas impermeable materials hinder the contact lens devices' use for continuous daily wear and potentially initiate corneal hypoxia and discomfort. Moreover, traditional materials lack optical transparency, mechanical compliance, and biocompatibility required for extended use. Advantageously, nanofiber hydrogels, which have similar physiological and mechanical properties to human tissue, can offer more comfort due to their softness and stretchability. Their high porosity and hydration level will facilitate the transportation of charges, ions, and molecules, which are crucial for contact lenses to retain moisture and gas permeability. Therefore, hydrogel contact lens electrodes, which use metal‐coated nanofiber meshes as electronic conductors and commercial hydrogel lenses as substrates, have been comprehensively investigated.^21^ They not only have high optical transparency but also represent outstanding mechanical compliance and durability. Surprisingly, they maintain high gas permeability, even when a large area of electronic conductors is required. Experimental results suggested that rabbit eyes wearing this hydrogel contact electrode for 12 h showed no evident sign of corneal wear or irritation, confirming its high safety during daily wear. Additionally, this device showed superior stability, high mechanical durability, and good wettability. Critically, a study using rabbit eyes has shown that this hydrogel contact lens electrode can be effectively used for electroretinogram (ERG) measurement, highlighting its significant advantages in terms of conformability, transparency, and potential patient acceptance. Furthermore, this electrode achieved stable contact with the cornea, thus maintaining a steady eye diopter with minimal corneal irritation. As subsequent versions may incorporate additional functionalities, it will be crucial to evaluate how such integration impacts the device's core properties, such as optical transparency, gas permeability, and mechanical compliance. We believe that after overcoming these difficulties, this advancement will create a versatile platform for seamless biointegration, and introduce wearable or implantable sensors with enhanced biocompatibility, thereby benefiting health monitoring and medical treatment.

### 6.5 Non‐infectious uveitis

Non‐infectious uveitis (NIU) is a significant cause of vision impairment, characterized by intraocular inflammation primarily attributed to autoimmune disorders.^91^ At present, diverse steroid drug preparations, such as eye drops, ointments, and injections, have been used to treat inflammatory reactions in uveitis. However, owing to limited intravitreous permeability and low bioavailability, these topical preparations often prove ineffective in managing the inflammatory reaction. In addition, while systemic administration of steroid drugs can cause severe adverse effects such as Cushing's syndrome, hyperglycemia, hypertension, peptic ulcer, infections, and ophthalmological disorders. Intravitreous injection of steroids is effective in the treatment of uveitis, but there is a higher risk of high intraocular pressure, cataract, retinal detachment and hemorrhage. In this context, a dexamethasone supramolecular hydrogel for intravitreal injection has been designed to realize the controlled release of steroid drug.^85^ This supramolecular hydrogel is fabricated by mixing an aqueous solution of Dex sodium phosphate (DexP) with an aqueous solution of CaCl_2_. The impact of Ca^2+^ concentration and Dex concentration on the supramolecular hydrogel's properties and the in vitro release dynamics has been studied in detail. A key advantage of this supramolecular system is its tunable release profile. The release rate of Dex could be precisely regulated by adjusting the concentration of Ca^2+^. Specifically, in PBS, at 37°C, increasing Ca^2+^ concentration from 2 to 4 mg/mL resulted in a measurable decrease in the cumulative drug release over 24 h (from ~70% to ~60%), demonstrating precise control over release profiles through ionic crosslinking density. In addition, to evaluate its efficacy, various in vivo anti‐inflammatory efficacy tests of Dex were performed on a rat model of experimental autoimmune uveitis (EAU). In vitro experimental results suggested that Dex supramolecular hydrogel had excellent thixotropic properties, and the release rate of Dex could be regulated by adjusting the concentration of Ca^2+^. Compared with traditional intravitreal injection drugs, the Dex supramolecular hydrogel did not cause side effects when the Dex content reached 30 μg/eye. Furthermore, the Dex supramolecular hydrogel could alleviate inflammation in the EAU model by down‐regulating Th1 and Th17 effect responses. Accordingly, this calcium ion‐coordinated Dex supramolecular hydrogel. In summary, this Dex supramolecular hydrogel not only possesses exceptional mechanical properties but also has satisfactory biocompatibility (Table 2). All these results conclude that the nanofiber hydrogel may be a promising therapeutic strategy for non‐infectious uveitis.

### 6.6 Self‐delivery system for ophthalmic drugs

Formulations of drug drops are currently the most common and acceptable route for treating eye diseases. However, due to poor corneal permeability and rapid precorneal drug clearance, less than 5% of the drug can reach the aqueous humor after local administration. Thus, frequent administration is required to maintain an effective therapeutic drug concentration. This results in large variation in the concentration of aqueous humor over time, which greatly increases the risk of adverse reactions. Innovatively, hydrogel‐controlled release drug represents a viable therapeutic alternative for the treatment of ocular diseases due to its advantages of easy loading and prolonged corneal retention. In contrast to the conventional drug delivery approaches, prodrug supramolecular hydrogel forms its own delivery system, which not only overcomes the disadvantage of poor water solubility of the drug, but also significantly alleviates concerns about carrier‐related side effects. Researchers extracted a prodrug supramolecular hydrogel from succinated dexamethasone (Dex‐SA) conjugates through a pH hydrolysis strategy.^86^ XRD and FTIR analysis showed that the drug molecules were amorphously dispersed in the hydrogel matrix. Moreover, the lyophilized Dex‐SA supramolecule hydrogel demonstrated excellent stability in vitro without causing any significant hydrolysis of Dex‐SA. Even at −20°C it could be stored for up to 30 days. The lyophilized Dex‐SA supramolecule was rapidly converted into hydrogel within 30 min after dissolution in distilled water without affecting its pharmacological activity. After local instillation, it was observed that Dex‐SA supramolecular hydrogel had perfect ocular tolerance. Compared with DexP solution, Dex‐SA supramolecular hydrogel had sufficient viscosity when injected locally and retained longer on the cornea of rabbits. Advantageously, this prodrug supramolecular hydrogel exhibits potent thixotropy, and better bioavailability over conventional viscous formulation (Table 2). Therefore, prodrug supramolecular hydrogels offer an ideal platform for drug delivery, particularly in the treatment of ocular diseases.

## 7 CHALLENGES AND OUTLOOK

The clinical transformation of these biomaterials and fabricating systems still faces enormous challenges. A case in point is the NT‐503‐1 implant for dry AMD, which, despite demonstrating sustained anti‐VEGF delivery in preclinical studies, failed to outperform standard monthly injections in a Phase II trial (NCT03144999). This setback underscores fundamental translational barriers, including: (1) inherent limitations of animal models in recapitulating human disease complexity; (2) the high efficacy threshold set by existing therapies; and (3) difficulties in scaling up manufacturing while ensuring product consistency and stability.

**FIGURE 5:**
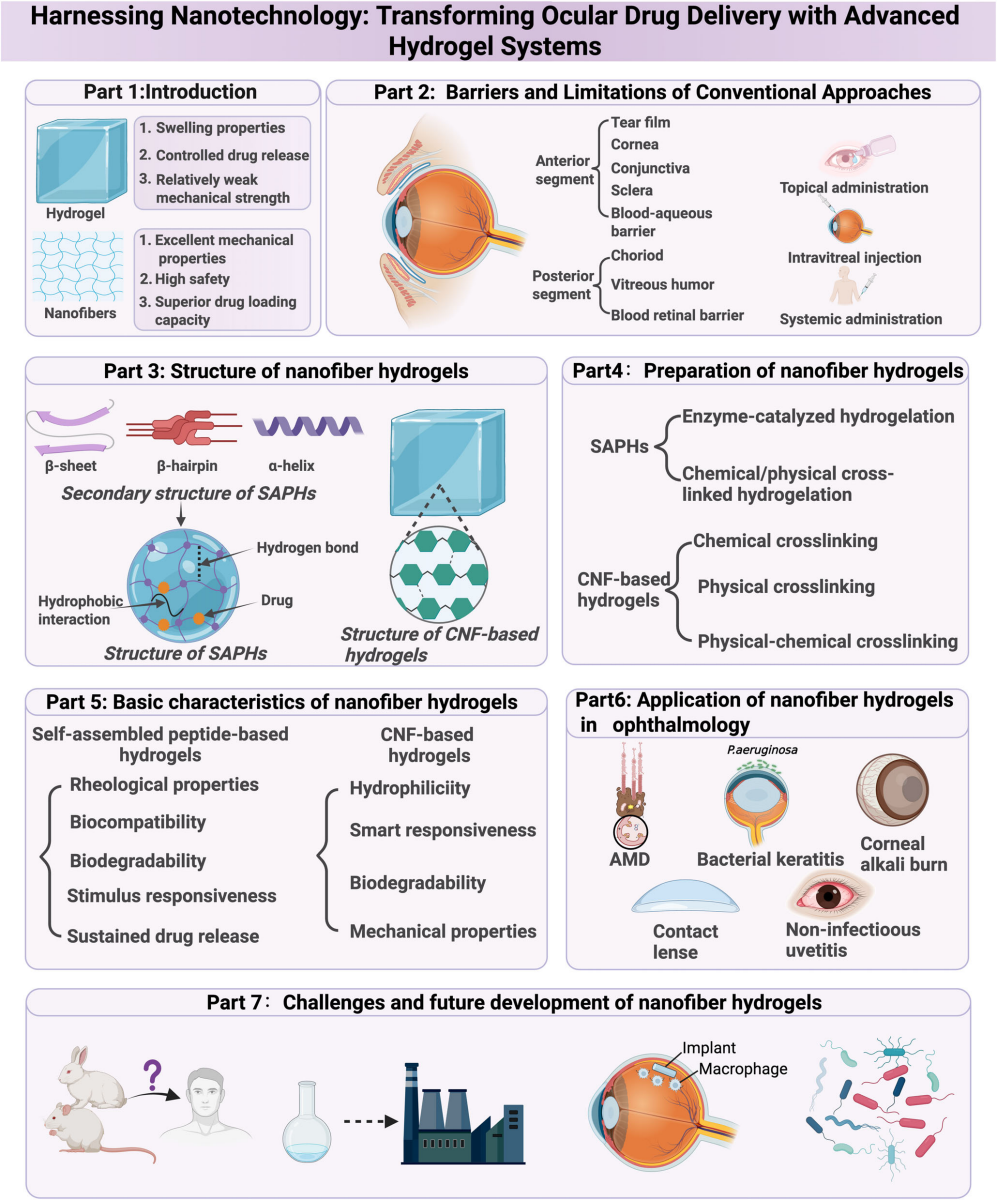
A summary diagram of this review. This figure is partitioned into seven integral sections, including introduction, barriers and limitations of conventional approaches, structure of nanofiber hydrogels, preparation methods of nanofiber hydrogels, fundamental characteristics, and ophthalmic applications, and challenges of nanofiber hydrogels. AMD, age‐related macular degeneration; CNF, cellulose nanofiber; SAPHs, self‐assembled peptide‐based hydrogels.

In addition to these general hurdles, ocular biomaterials confront unique anatomical and physiological challenges that must be squarely addressed. The long‐term biocompatibility of any material implanted in the eye is paramount. The ocular environment is highly immune‐privileged yet delicate. Meanwhile, chronic inflammatory responses to implant materials can cause vision‐threatening complications, including opacification and elevated intraocular pressure. For instance, the long‐term biocompatibility of anti‐VEGF@BetP and Dex supramolecular hydrogel mentioned in this article awaits further determination through experimental studies. Furthermore, optical clarity and refractive properties are indispensable for anterior segment applications such as the PUTK/RH patch or potential corneal implants. Any haze, turbidity, or irregular light scattering caused by biomaterials is functionally unacceptable and poses strict requirements for the purity of polymers, crosslinking density, and the uniformity of nanostructures. Finally, a major constraint lies in sterilization (Figure 5). The inherent sensitivity of many nanofiber hydrogels, particularly those composed of polypeptides or delicate polymers, renders them vulnerable to traditional terminal methods (e.g., autoclaving, gamma radiation, and EO exposure).^92^ The most effective sterilization methods (such as high temperature and strong irradiation) often have the greatest destructive effect on fragile nanostructures and active ingredients, while milder methods may not be able to ensure a sterile or pyrogen‐free state. Consequently, ensuring sterility often necessitates aseptic manufacturing or novel gentle technologies, which add substantial complexity and expense.

To facilitate the translation of nanofiber hydrogels to clinical applications, several key issues must be addressed. First, safety is the top priority. As highlighted previously, a comprehensive assessment of the biocompatibility of nanofiber hydrogels is critical, as their chronic safety profile in human patients has yet to be fully established. Second, the transition from laboratory‐scale fabrication to clinical and industrial‐grade production represents a central challenge in scalability (Figure 5). Overall, SAPHs face greater scalability hurdles due to peptide synthesis costs and self‐assembly control; CNF hydrogels present a more immediate path to scale‐up owing to their abundant raw materials and compatibility with existing industrial processes. Therefore, for both systems, the development of robust, efficient, and GMP‐compliant large‐scale production processes is a prerequisite for their clinical translation. Encouragingly, with the recent advances in nanofiber systems, the mass production of nanofibers will possibly meet the growing demands of population‐scale application.^93^ Third, material performance must be meticulously optimized. A critical aspect is achieving a balance between injectability and mechanical stability: the material should exhibit shear‐thinning behavior for easy administration through fine‐gauge needles yet rapidly recover sufficient strength post‐injection to prevent migration within the eye. Further essential objectives include precisely controlling the degradation rate to match therapeutic needs, enhancing drug‐loading capacity without sacrificing mechanical properties, and developing stimuli‐responsive systems for precise drug release. Currently, the clinical translation of nanofiber hydrogels faces complex regulatory hurdles that must be addressed throughout the development process. A primary obstacle is regulatory classification: since nanofiber hydrogels typically combine a structural scaffold (device) with a therapeutic agent (drug), they are considered combination products. Determining their primary mode of action (PMOA) is critical, as it dictates whether they will be regulated as drugs or devices—significantly influencing the development pathway. Furthermore, the safety profile of novel nanofiber components and degradation products requires extensive validation under GMP standards. Meanwhile, clinical trial design must demonstrate not only safety and efficacy but also clear advantages of the delivery system over existing treatments, such as reduced administration frequency for chronic conditions like AMD.^82^ Successfully navigating these regulatory challenges will be essential to realizing the full potential of nanofiber hydrogels in transforming ophthalmic drug delivery and therapeutics. Encouragingly, interdisciplinary collaborations are fostering innovations such as gene‐regulating nanofiber hydrogels for neural repair.^94^ With continued progress in manufacturing, safety evaluation, and regulatory planning, nanofiber hydrogels hold significant potential to expand therapeutic options in ophthalmology and beyond.

## 8 CONCLUSIONS

This review systematically discusses the versatile properties and fabrication methods of nanofiber hydrogels, highlighting their significant potentials in ophthalmology. Thus far, numerous nanofiber hydrogels have demonstrated their safety and efficacy for treating ocular diseases such as AMD, bacterial keratitis, ocular alkali burns, and non‐infectious uveitis. They also show promise in fabricating biomaterials for electronic smart contact lenses. However, prospective clinical studies are necessary to promote their translation. Future optimization of productive techniques, large‐scale evaluation of feasibility, and patient acceptability must be carefully considered to ensure successful clinical implementation. We believe that continuous exploration will enable more nanofiber hydrogels to achieve ultimate goals.

## AUTHOR CONTRIBUTIONS

**Huidi Cui**: Writing—review and editing, writing—original draft, methodology, conceptualization. **Hao Wu**: Writing—review and editing, writing—original draft, methodology, conceptualization. **Bizhu Zhao**: Methodology, formal analysis. **Yameng Zhao**: Formal analysis. **Chengzhi Zhang**: Formal analysis. **Mingxuan Wang**: Formal analysis. **Jiehao Zhang**: Formal analysis. **Kexin Wang**: Formal analysis. **Yiwen Wang**: Formal analysis. **Zongming Song**: Writing—review and editing, supervision, methodology. **Ye Tao**: Writing—review and editing, writing—original draft, supervision, conceptualization.

## CONFLICT OF INTEREST STATEMENT

The authors declare no conflict of interest.

## Data Availability

Data sharing not applicable to this article as no datasets were generated or analysed during the current study.
